# Effect of Occupation on Mortality
*Annual Report on the Health of the Parish of St. Marylebone. Year 1856. By Robert Dundas Thomson, M.D., F.R.S., Medical Officer of the Parish. London. 1857.


**Published:** 1858-04

**Authors:** 


### EFFECT OF OCCUPATION ON MORTALITY.*

Dr. R. D. Thomson, in his *Report on the Health of Marylebone in* 1856, has the following important notes on occupations and mortality.

"The influence of the peculiar every day duties in which the population are engaged, upon the relative duration of life, is a sanitary question of great import, in reference, not to the inevitably injurious effects of a profession, but to the unnecessary hurtfulness of various parts of occupations as now conducted, but which are susceptible of amelioration. The mortality among blacksmiths in St. Marylebone was as high as 31 per 1000 in 1856; while, in 1851, it was 19 in all England—the total mortality of

* Annual Report on the Health of the Parish of St. Marylebone. Year 1856. By Robert Dundas Thomson, M.D., F.R.S., Medical Officer of the Parish. London. 1857.

males at 20 and upwards, for the whole country, being 20 per 1000. Horsekeepers, jockeys, and grooms died at the rate of 35 per 1000. It becomes a natural inquiry, is this a peculiarity pertaining to the parish, or is it accidental, at the present period? Future observations will tend to throw light on the question; the mortality for the whole of London in 1851, among this class, was 23 per 1000. Shoemakers were affected with a mortality of 17 per 1000, that for the metropolis in 1851, being 18 per 1000; domestic servants had a death-rate of 13 per 1000, the average being 18 for London, showing the favourable position of this class in the parish. The mortality of tailors was 23; of carpenters, 21; of butchers, 20; of bakers, 9, against an average of 18; but there is obviously a great risk in drawing general deductions from the limited data afforded in a single locality, as to the nature of an occupation. More extended results must be compared, extending over a greater period of time." (pp. 5-6.)

